# Review of 107 Oncoplastic Surgeries Using an Acellular Dermal Matrix with the Round Block Technique

**DOI:** 10.3390/jcm11113005

**Published:** 2022-05-26

**Authors:** Hong-Il Kim, Byeong-Seok Kim, Yoon-Soo Kim, Hyung-Suk Yi, Jin-Hyung Park, Jin-Hyuk Choi, Sung-Ui Jung, Hyo-Young Kim

**Affiliations:** 1 Department of Plastic and Reconstructive Surgery, Kosin University Gospel Hospital, Kosin University College of Medicine, Busan 49267, Korea; immtkg4u@daum.net (H.-I.K.); seoook@naver.com (B.-S.K.); medissu@naver.com (Y.-S.K.); sencha21@naver.com (H.-S.Y.); atreyue@naver.com (J.-H.P.); 2 Department of Surgery, Kosin University Gospel Hospital, Kosin University College of Medicine, Busan 49267, Korea; drchoijinhyuk@gmail.com (J.-H.C.); ist2000good@daum.net (S.-U.J.)

**Keywords:** breast cancer, breast-conserving surgery, oncoplastic surgery, mammaplasty, breast reconstruction, acellular dermal matrix

## Abstract

The round block technique (RBT) is an oncoplastic surgery method that uses volume displacement techniques after partial mastectomy. However, cosmetic problems occur after tissue rearrangement in patients with small breasts or those in whom a large amount of breast tissue is excised. Therefore, we used an acellular dermal matrix (ADM) when the volume was insufficient after tissue rearrangement. Patients who underwent breast reconstruction using the ADM with the RBT after breast-conserving surgery (BCS) were included. The ADM graft was performed in two layers. First, it was placed on the glandular flap, and the patient was then seated to ascertain the degree of deformity. If the volume was insufficient, a graft was also performed under the skin flap. Overall, 107 oncoplastic surgeries were performed. Tumors were most commonly located in the upper outer quadrant of the breast, and the mean resected breast tissue was 27.1 g. Seroma was the most common complication, but it improved with several aspirations. There were no major complications or cosmetic problems requiring reoperation. Therefore, if the ADM was used for defects that could not be reconstructed with the RBT alone, safe and cosmetically good results could be obtained.

## 1. Introduction

In 1998, Audretsch et al. first attempted oncoplastic surgery, which is an expanded concept of breast-conserving surgery (BCS), and considered both tumor resection and aesthetics [1]. Oncoplastic surgery can be performed after BCS using either volume replacement or displacement techniques. The volume displacement technique is based on glandular reshaping or reduction mammoplasty, while the volume replacement technique uses autologous tissue for different types of flaps. The round block technique (RBT) is an oncoplastic surgery method that uses the volume displacement technique after partial mastectomy [2]. RBT is generally used when the tumor is close to the nipple, located on the upper or outer side, or when the resection volume is <100 g [3,4].

The breast size of Asian women is relatively small, whereas the tumor size is large [5,6]. Therefore, cosmetic problems, such as inframammary fold (IMF) retraction or depression deformity, occur after tissue rearrangement in patients with small breasts or in cases where a large amount of breast tissue is excised, especially in the lower and inner quadrants. Thus, it is difficult to achieve aesthetically satisfactory results using volume displacement with the breast tissue of the patient.

Here, we performed breast reconstruction using an acellular dermal matrix (ADM) when the volume was insufficient after tissue rearrangement. This study aimed to evaluate the safety, usefulness, and cosmetic results of the ADM.

## 2. Materials and Methods

### 2.1. Patients

In total, 107 patients aged 25–67 years with breast cancer who underwent BCS and oncoplastic surgery using the ADM with the RBT between March 2020 and March 2021 were enrolled. Regardless of tumor location, reconstruction surgery was recommended for patients who were expected to have a deformity after BCS and performed if the patients desired it. All patients were informed, and they consented to the possible use of the ADM during surgery. Patients underwent oncoplastic surgery using the ADM with the RBT by one plastic surgeon (HYK) after partial mastectomy with sentinel lymph node biopsies or axillary lymph node dissection in the Department of Breast Surgery. We recorded the patients’ demographic data, medical history, tumor type, location, resected breast tissue volume, and neoadjuvant chemotherapy, radiotherapy (RTx), and adjuvant chemotherapy administered.

All study participants provided written informed consent to store their medical information in the database and use it for research purposes. The study protocol was approved by the Institutional Review Board of Kosin University Gospel Hospital of Korea (KUGH 2021-09-040). All procedures were performed in accordance with the ethical standards of the institutional and national research committee and with the 1964 Helsinki Declaration and its later amendments.

### 2.2. Surgical Technique

First, the preoperative design was marked by a plastic surgeon. The incision line was determined differently depending on the tumor size and nipple–tumor distance; therefore, a periareolar incision or an outer circle within a distance from the periareolar circle of 1.5 cm was marked.

**Figure 1:**
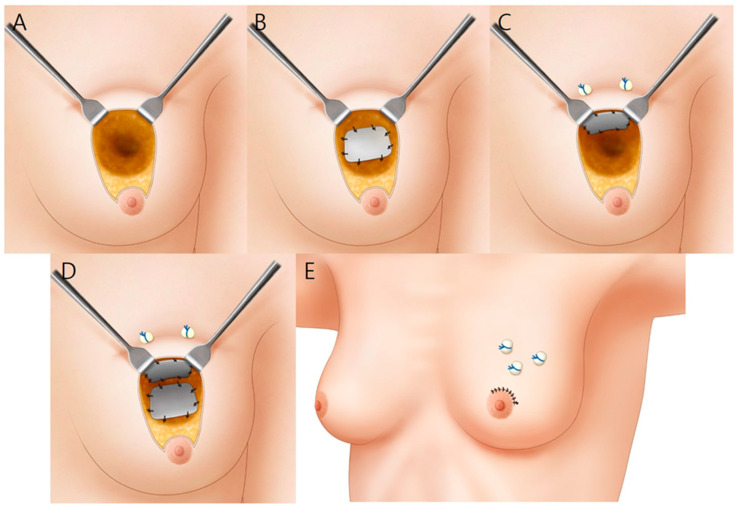
A schematic illustration summarizing the surgical procedure. (**A**) The glandular tissue is exposed after partial mastectomy; (**B**) An ADM is placed on the glandular flap and sutured; (**C**) An ADM is placed under the skin flap and fixed using the pullout suture technique with Prolene^®^ 2-0 sutures and bolsters; (**D**) The ADM graft is performed in two layers; (**E**) The suture is completed.

**Figure 2:**
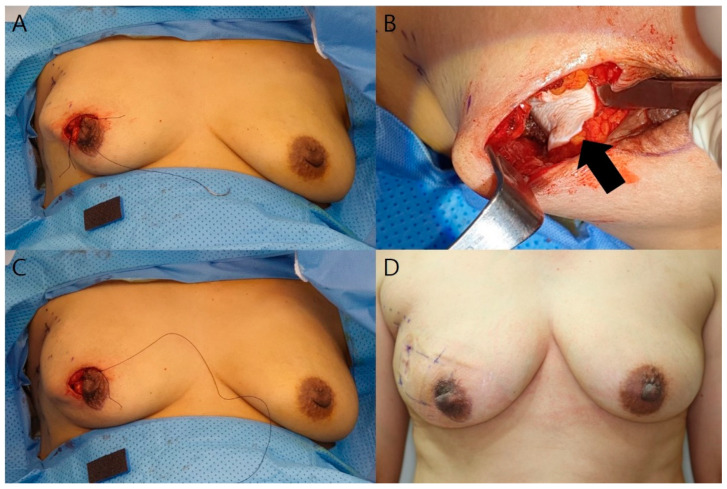
Intra- and postoperative photographs of a 52-year-old woman. The tumor is located in the upper outer quadrant of the right breast. The resected breast tissue was 50 g, and 2 sheets of ADM were used. (**A**) Intraoperative photograph after local tissue rearrangement. IMF retraction is also observed; (**B**) After the rearrangement is released, an ADM graft is placed on the pectoralis major muscle. The black arrow indicates the ADM; (**C**) After ADM graft placement, the IMF retraction disappears, and the breast volume is maintained; (**D**) Postoperative photographs after 1 month. The breast contour is maintained without any deformity.

The breast surgeon made the initial incision on the outer circle, and partial mastectomy with sentinel lymph node biopsy was performed (Figure 1A). Following this, the plastic surgeon performed the reconstruction. A glandular advancement flap was elevated from the pectoralis muscle from both sides according to the tumor location to cover the defect. A Jackson–Pratt (JP) drain was inserted into the subglandular area. Once the glandular flaps were approximated with Vicryl^®^ (Ethicon Inc., Somerville, NJ, USA) 1-0 sutures, IMF retraction or depression deformity might occur as the glandular flap was moved to the defect site (Figure 2A). When a deformity occurred, we removed the previous sutures, and then the flap was sutured in a different direction, and less approximated region and the area where the defect remained was covered with the ADM using Vicryl^®^ 1-0 sutures (Figure 1B and Figure 2B). The patient was then seated to ascertain the degree of depression (Figure 2C). Next, if the volume was insufficient, an ADM graft was also performed under the skin flap using the pullout suture technique with Prolene^®^ (Ethicon Inc., Somerville, NJ, USA) 2-0 sutures and bolsters to fix it (Figure 1D). Alternatively, if the skin flap remained thin, the ADM graft was performed under the skin flap only, and the glandular flap sutures were left as is (Figure 1C). In addition, when the breast volume was small, the amount of mass resection was small, or there was a defect in the lower pole, the ADM graft was performed immediately without glandular flap rearrangement. A subcuticular dermal running suture was performed with Monosyn^®^ (B. Braun, Melsungen AG, Melsungen, Germany) 4-0 suture, and a skin suture was performed with Nylon 5-0, and Steri-Strip^®^ (3M, St. Paul, MN, USA) was applied. A JP drain was placed for an average of 6 days, and if the daily drainage was <10 mL, the drain was removed, and the patient was discharged.

The ADM used for surgery was a CGCryoDerm^®^ (CGBIO Inc., Seongnam, Korea) cross-linked human ADM. It is a 3 × 4-cm square with a thickness of 2–3 mm. The ADM was melted in normal saline for 5 min in an operating room.

### 2.3. Satisfaction Survey and Complications

We retrospectively reviewed patient data to investigate complications during 12 to 18 months of follow-up; clinical photographs were used to evaluate the cosmetic results. A patient satisfaction survey and cosmetic evaluation by 2 board-certified plastic surgeons (H.I.K and H.Y.K) were performed postoperatively at 12 months. Patient satisfaction surveys were conducted based on BREAST-Q reconstruction modules version 2 [7]. The cosmetic evaluation was assessed by Aesthetic Items Scale (AIS) on a 5-point Likert scale (“very dissatisfied,” “dissatisfied,” “neutral,” “satisfied,” and “very satisfied”) using clinical photographs [8].

The data were analyzed using Microsoft Excel version 16.53 (Microsoft Corp., Redmond, WA, USA), and the Student’s *t*-test or the Kruskal–Wallis test was used to compare continuous variables, while the Pearson chi-square test was used to compare categorical variables. Statistical analysis was performed using IBM SPSS version 26 (IBM Corp., Armonk, NY, USA), and statistical significance was set at *p* < 0.05.

## 3. Results

### 3.1. Patients’ Characteristics

During the study period, 107 patients underwent oncoplastic surgery: one ADM was used in 57 patients, and two ADMs were used in 50 patients. The mean patient age was 48.3 years and the mean follow-up period was 15.1 months. Tumors were most commonly located in the upper outer quadrant of the breast, and the mean resected breast tissue was 27.1 g. The patients’ baseline characteristics and operative data are presented in Table 1.

### 3.2. Satisfactory and Cosmetic Outcome Evaluation

**Figure 3:**
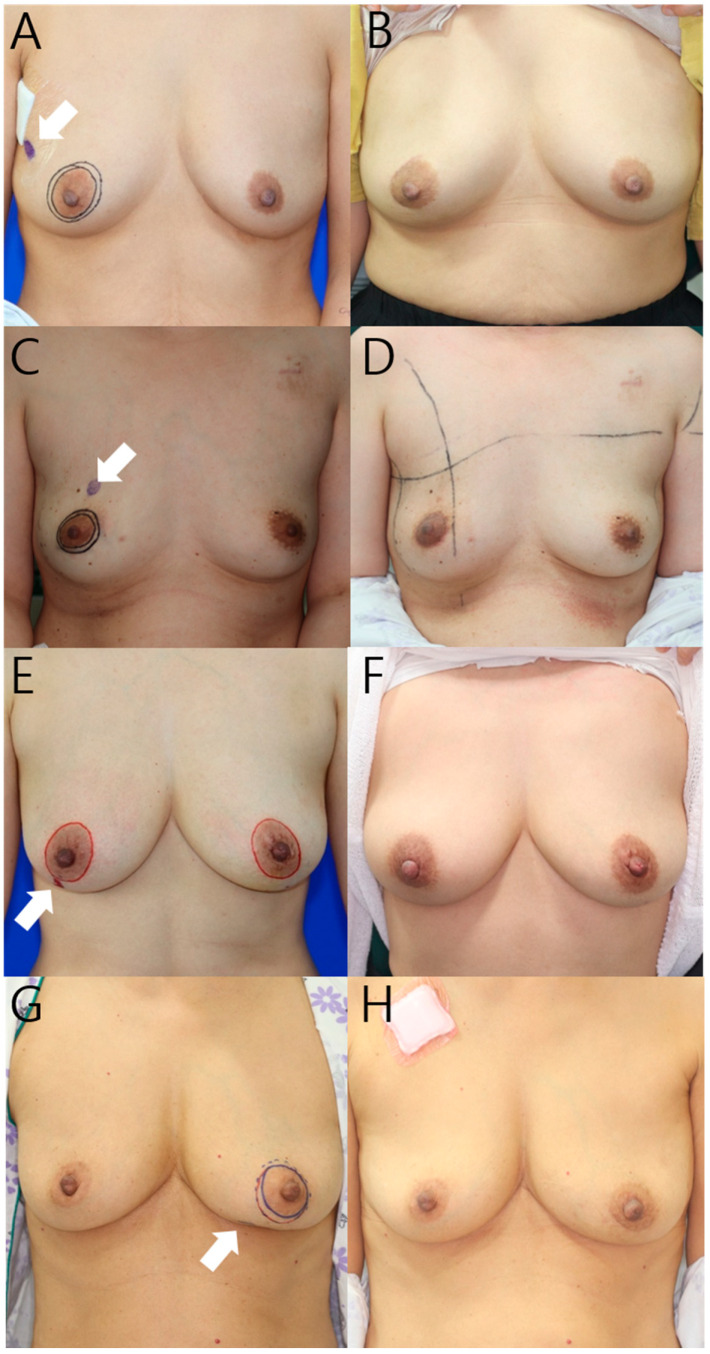
Preoperative (**A**,**C**,**E**,**G**) and postoperative (**B**,**D**,**F**,**H**) 12-month photographs listed by tumor location. White arrow indicates tumor location. (**A**) This 52-year-old patient’s tumor is located in the upper outer quadrant of the right breast, and the resected breast tissue was 22 g, and one ADM was used; (**C**) This 40-year-old patient’s tumor is located in the upper inner quadrant of the right breast, and the resected breast tissue was 21 g, and one ADM was used; (**E**) This 44-year-old patient’s tumor is located in the lower outer quadrant of the right breast, and the resected breast tissue was 28 g, and 2 sheets of ADM were used; (**G**) This 64-year-old patient’s tumor is located in the lower inner quadrant of the left breast, and the resected breast tissue was 18 g, and 2 sheets of ADM were used.

Twelve months postoperatively, a satisfaction questionnaire was administered to the patients, and two plastic surgeons evaluated the cosmetic score using clinical photographs. The mean satisfaction score was 84.0 (±9.3) in the patient group, and the mean AIS was 4.7 (±0.6) in the surgeon group (Table 2). Satisfactory and cosmetic outcomes revealed that >95% of the patients were satisfied. Regardless of tumor location, reconstruction was successful without any contour deformity (Figure 3).

### 3.3. Postoperative Complication Data

**Figure 4:**
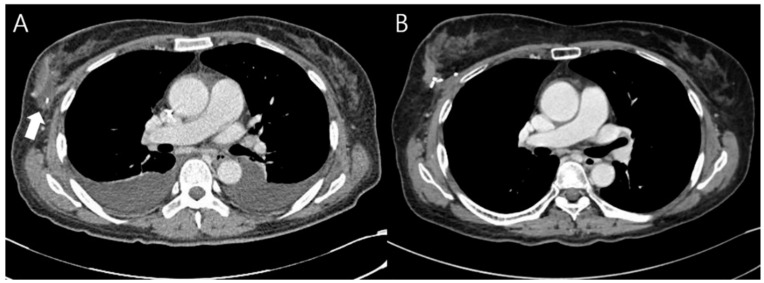
Chest CT image 3 weeks and 13 months after surgery. (**A**) A 54-year-old patient underwent breast reconstruction using the one ADM with the RBT after breast-conserving surgery. At 3 weeks after surgery, she complained of dyspnea during chemotherapy and a chest CT was taken. Seroma was noted on the chest CT (white arrow), and 5cc aspiration was performed, and was not observed when follow-up aspiration was performed 1 week later; (**B**) Seroma was no longer observed on the chest CT taken at 13 months after surgery.

A total of 35 patients (32.7%) developed postoperative complications during the follow-up period (Table 3). Seroma was the most common complication, but it improved with several aspirations in all patients. This was evaluated using physical examination and ultrasonography. There was no volume change during the follow-up period after aspirations, and it was confirmed that no seroma remained on computed tomography (CT) and ultrasonography performed 12 months after surgery (Figure 4). Seroma occurred in 12 patients in the one-ADM group (21.1%) and 21 in the two-ADM group (42.0%). The incidence according to ADM counts was statistically significant in the chi-square test (*p* = 0.037). In the two-ADM group, the odds ratio was 3.95. Hematoma occurred in one patient and was liquefied after two subcutaneous injections of hyaluronidase (1.5 mL) at 1-week intervals. Infection occurred in one patient and was resolved without removal of the ADM after the administration of cefazedone 2 g twice a day for a week postoperatively.

**Figure 5:**
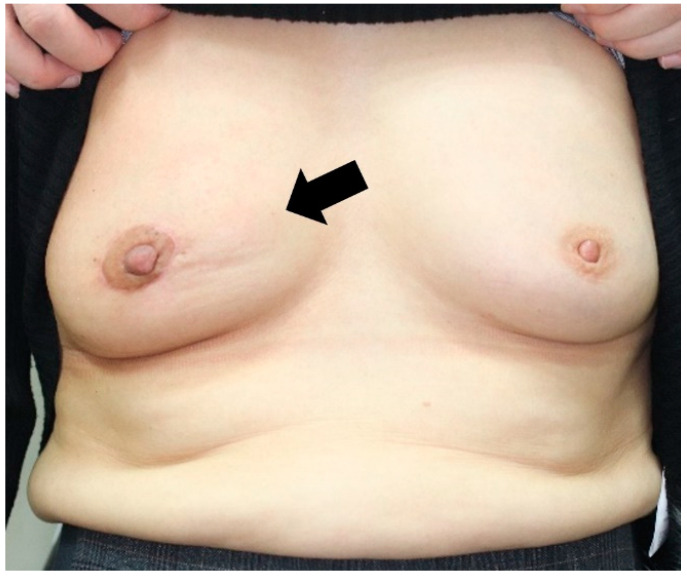
A 56-year-old patient’s 14-month postoperative photograph. The tumor was located in the upper inner quadrant of the right breast. And the resected breast tissue was 10 g, and 2 sheets of ADM were used. The black arrow indicates bulging deformity with severe hardness.

Only five patients (4.6%) developed cosmetic problems during the follow-up period. Depression deformity was observed in four patients (3.7%), and no additional surgical intervention, such as fat grafting was performed. A bulging deformity was observed in one patient (0.9%) (Figure 5). Thus, there were no major complications or cosmetic problems requiring reoperation.

## 4. Discussion

This review suggests that breast reconstruction using the ADM with the RBT is relatively feasible and cosmetically satisfactory in any breast quadrant.

### 4.1. Oncoplastic BCS

With a better understanding of the biological behavior and history of breast cancer, BCS is now widely accepted as the standard treatment for early breast cancer. As BCS has rapidly gained popularity, breast surgeons have realized that BCS might result in a breast deformity worse than that after total mastectomy in some patients [9]. In general, resection of >20% of breast tissue is a risk factor for deformity development [10]. In the case of the upper inner quadrant and lower pole, there is a risk of deformity even if <20% of breast tissue is resected [11].

Oncoplastic BCS has emerged to address these problems as a third option between conventional BCS and mastectomy, extending the BCS role, where there is a conflict of interest between wider excision and cosmetic outcome [12]. This can be performed using either volume replacement or displacement techniques. The volume displacement technique involves breast tissue advancement, rotation, or transposition using a breast flap or reduction technique [3]. Studies have examined volume replacement approaches in oncoplastic BCS, such as fat grafts or filling ADMs in a dice shape at the defect site [13,14]. We were able to fill the volume evenly by grafting a 3 × 4 cm ADM into two layers, and the complication rate was also low compared to those in similar studies using the ADM [14]. Angiogenesis and extracellular matrix (ECM) deposition are required for the ADM to be obtained; however, if the ADM is stacked in a square shape or rolled up in a cylinder, blood may not be supplied to the inner portion of the ADM for the same period; thus, it may not be obtained well [15]. Therefore, we ensured that the dermis layer had as much contact as possible with the part with blood flow. The ADM was spread and fixed on the glandular flap, and, if necessary, a two-layer graft of the ADM was performed to cover the defect. During the follow-up period, the ADM was obtained without volume loss or reconstruction failure. In addition, no additional surgery was performed for local recurrence during the average follow-up period of 15.2 months.

### 4.2. Surgical Method

In western women with excised volume measuring less than 20%, satisfactory cosmetic results can be achieved with glandular reshaping, and in the case of small to moderate breast sized Asian women, the patient who underwent glandular tissue reshaping with the ratio of the tumor specimen weight to breast volume of 0.12 g/mL (mean excise breast weight; 40.46 g) showed cosmetically satisfactory results [16,17]. In particular, the RBT is generally used when the tumor is close to the nipple, located on the upper or outer side, or when the resection volume is <100 g; however, a recent study reported good cosmetic results regardless of tumor location [3,4,18]. Our method can also be performed in all locations, and an ADM graft is performed when the breast volume is small or when it is difficult to maintain the contour with only local tissue rearrangement in intraoperative findings (e.g., IMF retraction, depression deformity). In most cases, the superior glandular flap was dissected and sutured, which was reduced as much as possible, and the glandular tissue was rearranged with Vicryl^®^ 1-0 sutures, and an ADM graft was performed when deformity occurred. When there was not enough tissue remaining for glandular tissue rearrangement and when the skin flap was thin, especially inner and lower pole breast, only the ADM graft was performed. The ADM was received well during the follow-up period without volume loss, and the contour was preserved; therefore, we recommend using the exact size of the ADM.

### 4.3. Complications

The most common complication was seroma (11.2%), which occurred during angiogenesis and ECM deposition [15]. All seroma complications were resolved with 2–3 aspirations without reoperation; only one patient required aspiration once a week for a month. It was found that seroma occurred in 6.5% of RBT reconstruction without the ADM in another study; therefore, it was confirmed that a large number of seromas occurred when the ADM was used [18]. When two ADMs were used, the incidence of seroma was significantly higher than that when one ADM was used. In the case of using two ADMs, the authors do not think that seroma will necessarily occur as the two ADMs do not completely overlap, because the patient is seated and the area to be performed with the ADM graft is marked under the skin flap. Thus, seroma occurred in 42% of the two-ADM group, but all was resolved after several aspirations.

We did not observe red breast syndrome or reconstruction failure requiring reoperation, such as severe infection or fat necrosis.

### 4.4. ADM

The ADM contains collagen, elastin, proteoglycans, laminin, and a basement membrane. These materials mostly serve as pillars for re-epithelialization, neovascularization, and fibroblast infiltration within a month so the probability of seroma is reduced thereafter [15]. The ADM does not evoke an immune response and is widely used in burn care and reconstructive surgery [19]. In breast surgery, the ADM is used in >75% of immediate tissue expander reconstruction procedures to support the implant [20] and reduce complications, such as capsular contracture during implant reconstruction after total mastectomy, and is also used for volume replacement in oncoplastic surgery [14,20]. We used the CGCryoDerm^®^ because there have been reports of less capsular contracture and less infection compared to other ADMs [21]. Moreover, cross-linked ADM is widely used because of its characteristics of rapid host cell infiltration and ECM deposition [15].

### 4.5. Oncoplastic Surgery and ADM Usage

**Figure 6:**
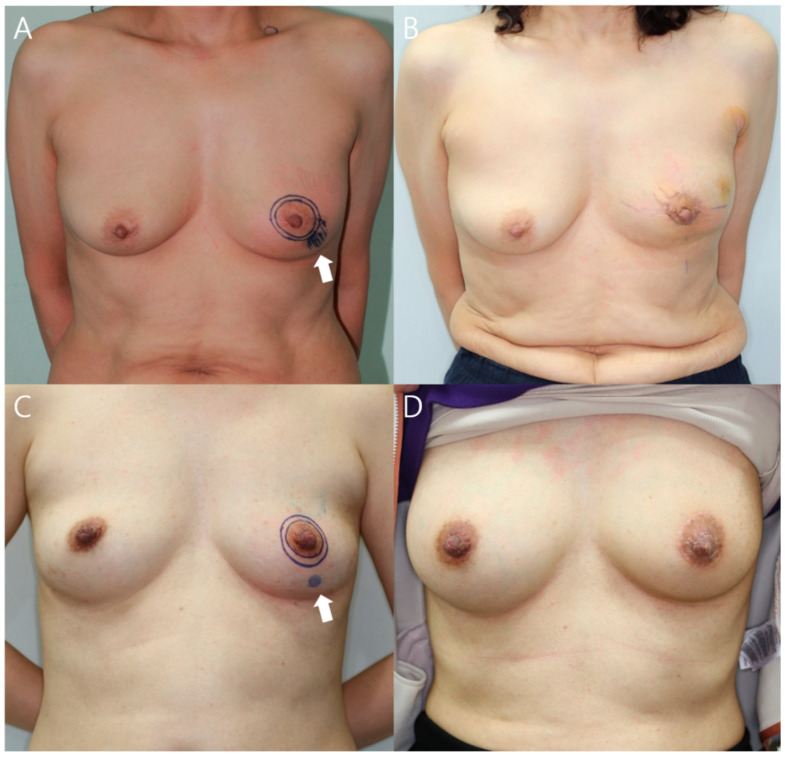
Preoperative (**left**) and postoperative (**right**) 12-month photographs of patients with cancer located in the lower outer quadrant of the left breast. White arrow indicates tumor location. (**A**) This 53-year-old patient underwent breast reconstruction with RBT alone after breast-conserving surgery and the resected breast tissue was 13 g; (**B**) Depression deformity was noted 12 months after surgery; (**C**) This 41-year-old patient underwent breast reconstruction using the one ADM with the RBT after breast-conserving surgery and the resected breast tissue was 12 g; (**D**) There were no cosmetic problems after 12 months of surgery.

During the study period, 158 reconstructions were performed after BCS; 68% of the patients used the ADM, and RBT reconstruction without the ADM was performed for the rest. In a previous study, there was no significant difference in the incidence of complications (present study, 13.0%, vs. the previous study, 11.1%), and cosmetic complications were much lower (5.6% vs. 15.7%) [18]. Additionally, although it was difficult to solve depression deformity during breast lower pole reconstruction with RBT alone, much better cosmetic results were obtained by performing an additional ADM graft (Figure 6). Thus, reconstruction using the ADM was performed with safe and cosmetically satisfactory results. The ADM graft was performed when lipofilling or flap surgery was rejected during the preoperative consultation. The authors thought that ADM, a solid supplement, was appropriate when considering donor site scar and fat resorption [22,23].

## 5. Conclusions

The strength of this study is that it presented objective results with considerable evidence, such as clinical photographs and experiences. In addition, we presented an ADM graft technique using a two-layer graft to maintain breast volume after BCS and showed successful engraftment rates. Therefore, the ADM could be used for defects that cannot be reconstructed with the RBT alone, thus providing safe and cosmetically good results without major complications.

## Figures and Tables

**Table 1:** Patients’ baseline characteristics and operative data.

Variables	Value *
Total number of patients	105
Total number of oncoplastic surgeries	107
1 ADM graft	57 (53.3%)
2 ADM grafts	50 (46.7%)
Age (year)	48.3 ± 8.3 (25–67)
BMI (kg/m^2^)	23.5 ± 2.9 (18.0–32.8)
Follow-up period	15.2 ± 1.9 (12–18)
Breast tumor quadrant	
Upper outer	51 (47.7%)
Upper inner	24 (22.4%)
Lower outer	22 (20.6%)
Lower inner	10 (9.3%)
Histological type	
Invasive ductal carcinoma	84 (78.5%)
Ductal carcinoma in situ	17 (15.9%)
Tubular carcinoma	3 (2.8%)
Mucinous carcinoma	2 (1.9%)
Invasive lobular carcinoma	1 (0.9%)
Mean resected breast tissue (g)	27.1 ± 17.4 (6–102)
0–50	89 (83.2%)
50–100	16 (14.9%)
>100	2 (1.9%)
Treatment	
Neoadjuvant chemotherapy	20 (19.0%)
Radiotherapy	104 (99.0%)
Adjuvant chemotherapy	58 (55.2%)

* Values are expressed as median (range) for continuous variables and number (percentage) for categorical variables. Abbreviations: ADM, acellular dermal matrix; BMI, body mass index.

**Table 2:** Satisfactory outcomes using the BREAST-Q and AIS.

Variables	Value *
BREAST-Q score	84.0 ± 9.3 (62–100)
Median AIS score	4.7 ± 0.6 (3.3–4.8)

* Values are expressed as median (range) for continuous variables. Abbreviations: AIS, aesthetic item scale.

**Table 3:** Postoperative complications and cosmetic problems.

Variables	Value *
Postoperative complications	35 (32.7)
Seroma	33 (30.8)
1 ADM graft	12 (21.1)
2 ADM grafts	21 (42.0)
Hematoma	1 (0.9)
Infection	1 (0.9)
Cosmetic problems	5 (4.6)
Depression deformity	4 (3.7)
Bulging deformity	1 (0.9)

* Values are expressed number (percentage) for categorical variables.

## Data Availability

Not applicable.
